# Effect of Processing Technology on the Degradation Behavior of Poly(p-dioxanone) Cog Threads

**DOI:** 10.1093/asjof/ojag073

**Published:** 2026-04-22

**Authors:** Duo Li, Qiufeng Wang, Kun Zhang

## Abstract

**Background:**

Poly(p-dioxanone) (PPDO) cog threads are widely used in facial rejuvenation. Common barb-fabricating techniques for PPDO threads include injection molding, compression molding, and cutting. These methods subject PPDO to distinct thermal and mechanical histories, potentially altering its internal structure. However, how these structural differences affect degradation and clinical longevity remains unclear and requires systematic investigation.

**Objectives:**

To investigate the influence mechanisms of 3 processing techniques on the *in vitro* degradation behavior and mechanical properties of PPDO cog threads.

**Methods:**

Poly(p-dioxanone) cog threads prepared by 3 techniques were degraded in 37°C PBS for 32 weeks. The mass loss rate, inherent viscosity, tensile strength, grasping force, and scanning electron microscopy (SEM) were measured periodically.

**Results:**

All samples exhibited a 2-stage degradation profile. In the first 8 weeks, mass loss was slow, inherent viscosity decreased rapidly, and tensile strength declined uniformly. After 8 weeks, mass loss accelerated, inherent viscosity reduction slowed, and tensile strength was largely lost. Injection-molded threads (highest processing temperature) degraded the fastest; unheated cut threads retained better molecular chain integrity for prolonged degradation; compression-molded threads had an intermediate degradation rate. For mechanical properties, cut threads had the slowest initial tensile strength loss due to no thermal damage (all 3 converged in later stages); injection-molded threads exhibited faster grasping force retention decline than compression-molded ones, confirming intensive thermal history accelerates degradation.

**Conclusions:**

The processing technology significantly influences the degradation rate of PPDO cog threads. Rational selection of processing methods can tailor degradation behavior, providing theoretical support for choosing the optimal cog threads in clinical applications.

**Level of Evidence: 5 (Therapeutic):**

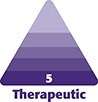

With advancing age, the human face undergoes a series of aging changes, including drooping of the malar fat pad, deepening of nasolabial folds, loss of jawline definition, and the appearance of localized volume deficits. These changes significantly compromise facial aesthetics and can diminish self-confidence. Poly(*p*-dioxanone) (PPDO) is a polymer widely used in medical fields, such as surgical sutures and facial implant threads, owing to its favorable mechanical properties, flexibility, biodegradability, and biocompatibility.^1-3^

Poly(*p*-dioxanone) cog threads are the most common type of facial implant threads in medical aesthetics, functioning by suspending and anchoring tissue.^4^ Furthermore, during degradation process, they can stimulate collagen production in the surrounding tissues,^5^ thereby helping to maintain and prolong the rejuvenating effects. Their evolution had focused on enhancing lifting capability. The earliest cog threads were manufactured using a cutting process, with barbs directly cut on the smooth monofilament (as shown in Figure 1). However, this technique often compromises the filament's core structure, potentially weakening its tensile strength and posing a risk of breakage during tissue lifting. To address this, compression-molded and injection-molded threads were developed. The compression molding process involves softening the raw PPDO thread at a temperature below its melting point and then pressing it into a mold under pressure. After cooling and solidification, the excess material is trimmed away, resulting in a monolithic, single-material cog thread (as shown in Figure 2). In contrast, the injection molding process begins by feeding PPDO pellets into an injection unit, where they are heated above their melting point. The molten PPDO is then injected into a closed mold, encapsulating a central core thread that passes through it. Upon cooling, this forms a cog thread with a distinct sheath–core structure, where the molded barbs coating on the central core (as shown in Figure 3). Both compression molding and injection molding eliminate damage to the core thread and allow for the creation of larger barbs, resulting in more reliable lifting performance.

**Figure 1.**
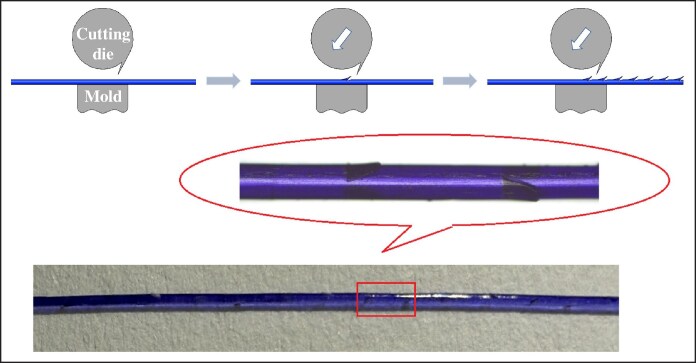
Principle of cutting process.

**Figure 2.**
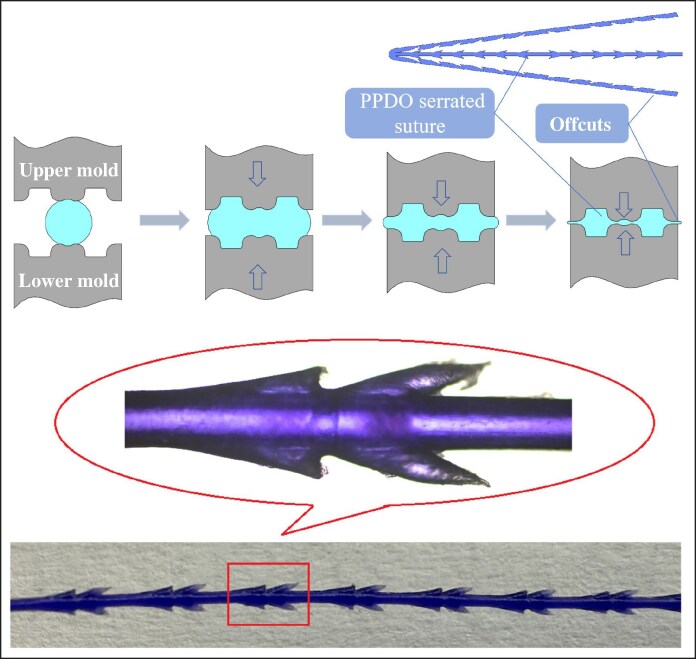
Principle of compression molding process. PPDO, Poly(p-dioxanone).

**Figure 3.**
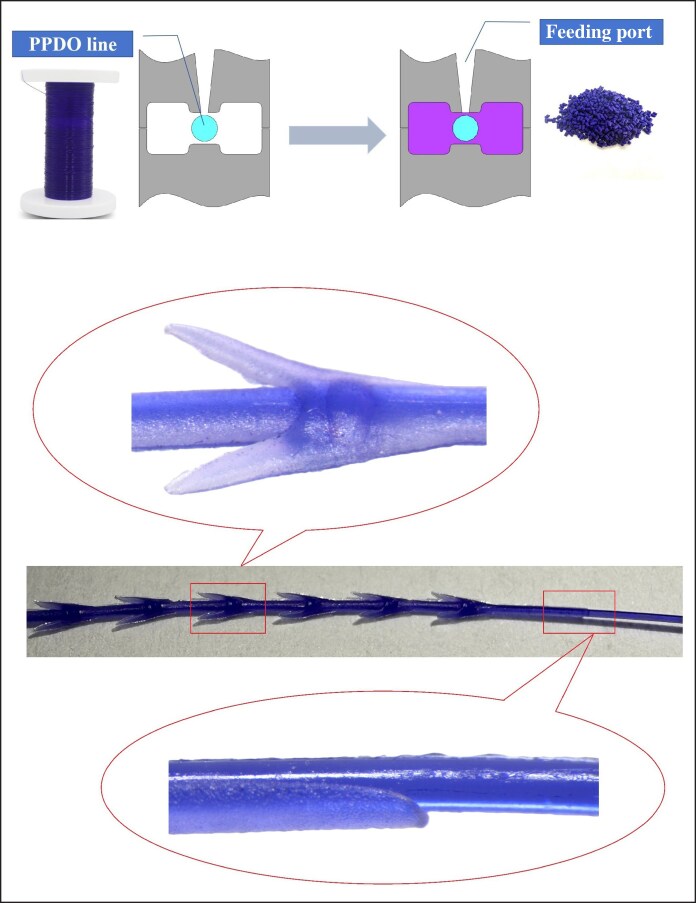
Principle of injection molding process. PPDO, Poly(p-dioxanone).

One of the most important properties of PPDO cog threads is their biodegradability. The degradability of PPDO stems from the ester bond structure within its molecular chains, which enables it to undergo biodegradation through hydrolysis.^6^ However, this degradation characteristic is a double-edged sword, presenting both significant advantages and certain challenges. On the one hand, hydrolysis allows PPDO to degrade completely in the physiological environment, producing small-molecule byproducts that are nontoxic and free of side effects.^7^ This eliminates the need for secondary surgical removal and ensures excellent biocompatibility. On the other hand, as hydrolysis progresses, the mechanical and physicochemical properties of the material undergo changes.^8,9^ If the hydrolysis rate does not align with the process of tissue repair and regeneration, the premature loss of mechanical properties may occur, thereby compromising the product's efficacy and long-term stability. Therefore, controlling and evaluating the degradation behavior of PPDO materials holds significant clinical importance.

Currently, there is a lack of research on the degradation behavior of the cog threads produced by different processing techniques, making it impossible to predict the differences in clinical lifespan and efficacy among threads made by various processes. Therefore, this study takes PPDO cog threads manufactured by 3 primary processing techniques currently available in the market—injection molding, compression molding, and cutting—as the research subjects. Systematic *in vitro* degradation experiments are conducted under conditions simulating the internal human environment (at 37°C, in phosphate buffer solution). By periodically monitoring the dynamic changes in key indicators such as mass, mechanical properties (tensile strength and grasping force), and molecular weight (represented by inherent viscosity) during the degradation process, this research thoroughly compares and analyzes the effects of different processing techniques on the degradation behavior of PPDO cog threads. The study aims to reveal the relationship between processing techniques and degradation performance, providing a scientific basis for clinicians to select the appropriate cog threads based on different facial lifting needs, while also offering theoretical guidance for the optimization of production processes and the precise control of performance in PPDO thread materials.

## METHODS

### Materials

Three types of PPDO cog threads with distinct processing technologies were in-house manufactured, all of which are similar to the 2-0 gauge specification of the smooth lines and feature a degradation period of 6 to 8 months.

### Methods

The degradation properties of PPDO have been reported in existing studies. Ray investigated the *in vivo* and *in vitro* degradation properties of PPDO fibers, and the results showed that the mass degradation rates were basically consistent in both systems, with nearly identical changes in mechanical properties.^10^ This finding provides a crucial insight for researchers: *in vitro* degradation assays can be used to simulate and predict the *in vivo* degradation behavior of PPDO. Based on this, Li further explored the *in vitro* degradation properties of PPDO in different media.^11^ They selected distilled water (pH = 6.50), normal saline (pH = 4.50), and phosphate-buffered saline (PBS, pH = 7.44) as the degradation media and conducted a degradation study on PPDO samples at 37°C. The results indicated that the degradation rate of PPDO samples was significantly accelerated in distilled water and normal saline. In addition, numerous scholars have carried out extensive research on the degradation properties of PPDO in PBS, and the variation trends of mass, mechanical properties and molecular weight of PPDO during degradation have been confirmed, with a degradation cycle of 6 to 8 months.^12-14^ Based on the above research findings, we designed an *in vitro* degradation test of 32 weeks duration in PBS at 37°C.

Poly(p-dioxanone) samples were fully immersed in PBS buffer (pH = 7.4) and placed in a 37°C stability test chamber. During the test period, the pH value of the test solution in the container should be monitored as needed. If the buffer pH deviates beyond the range of 7.4 ± 0.2, it should be adjusted using 0.1 mol/L sodium hydroxide solution or dilute phosphoric acid, or replaced to meet the requirements. For each type of cog threads, 100 samples were fabricated at a ratio of 0.1 g cog threads to 50 mL PBS buffer.

The PBS (pH 7.4) was prepared by mixing 18.2% solution A and 81.8% solution B (by volume). Sodium azide should be added to the PBS at a mass fraction of 0.02%. Solution A was 1/15 mol/L potassium dihydrogen phosphate solution, and solution B was 1/15 mol/L disodium hydrogen phosphate solution.

The degradation period of PPDO fibers is 6 to 8 months. Designed degradation sampling points were set at 2W, 4W, 8W, 16W, 20W, 26W, 30W, and 32W, with the 0-day sample serving as the reference. For samples at each sampling point, mass, tensile strength, grasping force, and intrinsic viscosity were sequentially measured. Each test item requires 6 replicate tests.

SPSS was used to perform a repeated-measures variance (ANOVA) (*α* = 0.05) to examine whether there were significant differences in the degradation rates of cog threads under different processing technologies.

### Mass Loss Rate

The degraded samples were rinsed with water. The rinse solution was then added to the degradation solution. The mixture was filtered through preweighed 0.2 μm filter paper. After drying the filter cake at 50°C to constant mass, the mass loss rate was calculated based on the initial sample mass (*m*_0_), the mass of the filter paper (*m*_2_), and the mass of the filter paper with the residue (*m*_1_). Mass loss rate = [1 − (*m*_1_ − *m*_2_)/*m*_0_] × 100%.^15^

### Intrinsic Viscosity

The dried samples from 2.2.1 were used for intrinsic viscosity measurement. The 0.025 g sample was dissolved in hexafluoroisopropanol at 25°C ± 1°C to obtain a 0.001 g/cm3 solution. Using an Ubbelohde viscometer (capillary diameter 0.4-0.5 μm), efflux times for the pure solvent (*T*_0_) and polymer solution (*T*) were measured.


$$\mathrm{Intrinsic}\;\mathrm{viscosity}\;=\;\ln\;(T/T_{0})/c.$$

### Tensile Strength

The degraded samples were taken and wiped on the surface until no water droplets remained. The distance between the fixtures of the electronic universal testing machine was set to 130 mm ± 5 mm, with a crosshead speed of 300 mm/min. Both ends of the thread were secured to the fixtures of the testing machine, ensuring the thread was taut. The test was then conducted, and the maximum force value was recorded.

### Grasping Force

**Figure 4.**
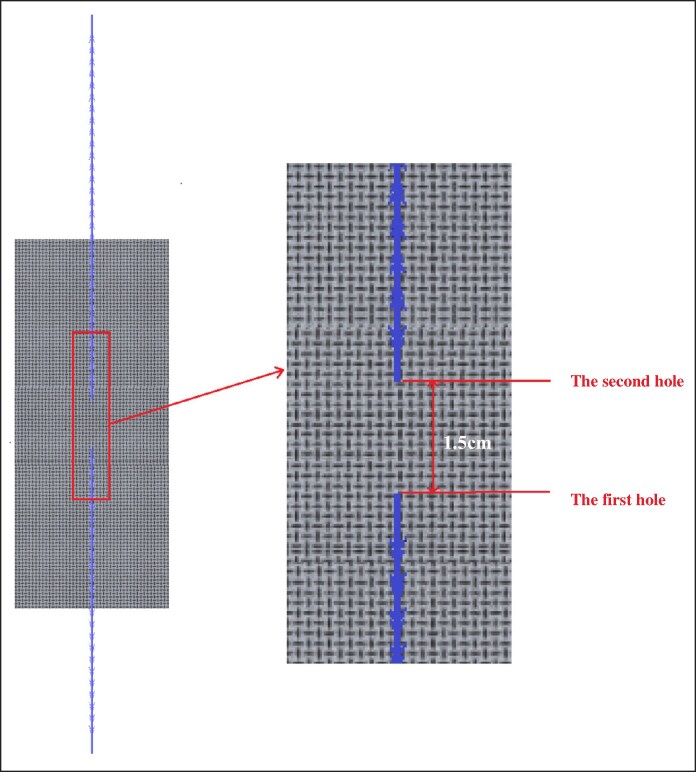
Sample preparation for grasping force.

A coarse filter mesh (G4 grade, 5 mm thick) was cut into 6 cm × 2.5 cm pieces. The cannula was passed vertically through the filter mesh and then reversed, creating 2 holes spaced 1.5 cm apart. After withdrawing the cannula, the cog thread was left in the mesh, and the first or second barb of the lower half of the thread was adjusted to be positioned at the second hole (as shown in Figure 4).

**Figure 5.**
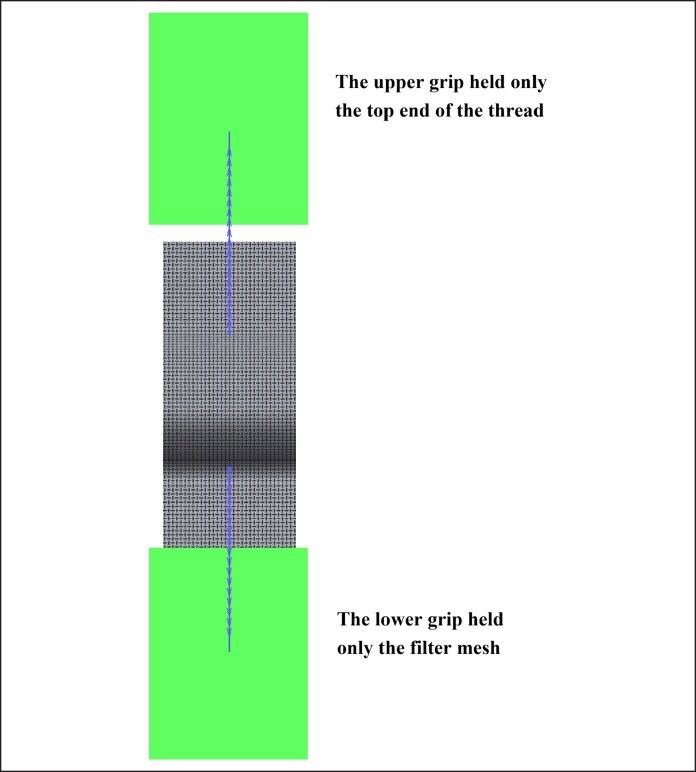
Testing setup.

The mentioned assembled samples were installed onto the fixture of the electronic universal testing machine, with the upper grip holding the thread and the lower grip holding the filter mesh (as shown in Figure 5). The distance between the fixtures was set to 6 cm. The thread was then pulled out against the barb orientation at 45 mm/min, and the maximum force was recorded.

## RESULTS

### Mass Loss Rate

**Figure 6.**
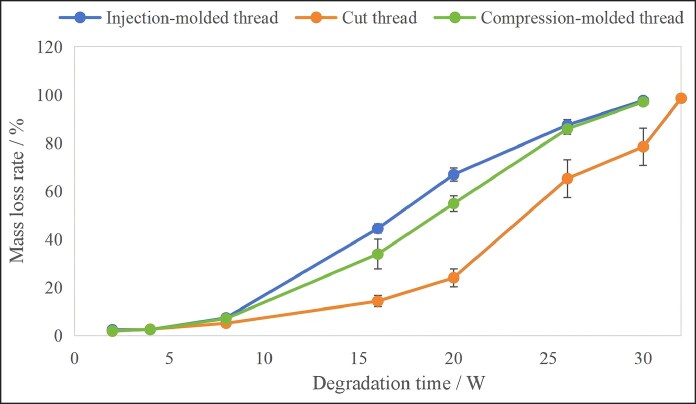
Mass loss rates of the cog threads with different processing techniques during degradation (*n* = 6).

The mass loss results of the cog threads manufactured by different processing techniques during degradation are shown in Figure 6. All 3 types of samples exhibited a typical 2-stage degradation: mass loss was slow (<10%) in the first 8 weeks, followed by a rapid degradation phase after 8 weeks. At 30 weeks, both injection-molded and compression-molded threads had reached a mass loss rate exceeding 95%, indicating that the degradation endpoint had essentially been attained.

Overall, repeated-measures ANOVA revealed that the mass loss rates of the 3 cog threads were highly significantly different, with a *P*-value of 0.000 in the between-subjects effects test, which is <0.05. This indicates the injection-molded thread degraded faster. This may be related to the higher heating temperature during barb formation. Higher processing temperatures are known to promote the degradation of the final product. In contrast, the cut thread only had a mass loss of 78.5% at 30 weeks. It reached a mass loss rate over 95% at 32 weeks, which was identified as its degradation endpoint.

### Retention of Intrinsic Viscosity

**Figure 7.**
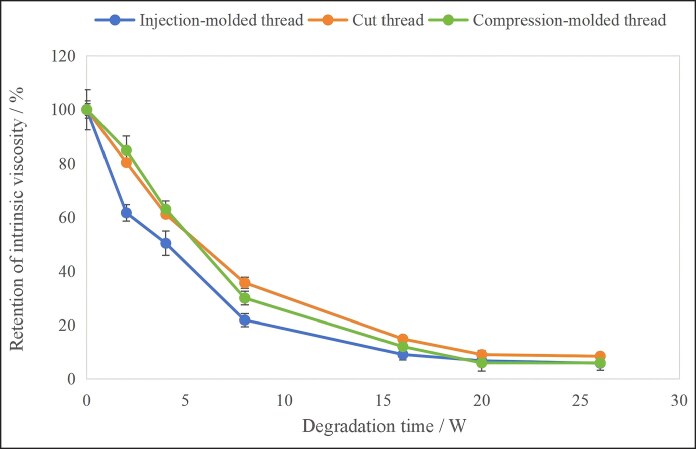
Retention of intrinsic viscosity for the cog threads with different processing techniques during degradation (*n* = 6).

Since intrinsic viscosity has a quantitative relationship with polymer molecular weight, it is commonly used to characterize molecular weight. Figure 7 shows the retention of intrinsic viscosity for the cog threads produced by different processing techniques during degradation. All 3 samples exhibited a rapid decrease in intrinsic viscosity within the first 8 weeks, after which the decline slowed considerably. This indicated that the reduction in molecular weight occurred primarily during the initial 8-week period.

Repeated-measures ANOVA revealed that the retention of intrinsic viscosity of the 3 cog threads were highly significantly different, with a *P*-value of 0.000 in the between-subjects effects test, which is <0.05. This indicates the injection-molded thread degraded faster.

### Retention of Tensile Strength

**Figure 8.**
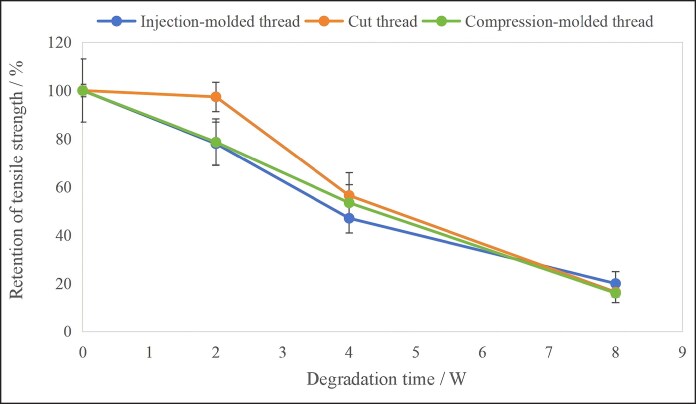
Retention of tensile strength for the cog threads with different processing techniques during degradation (*n* = 6).

Maintaining adequate tensile strength is fundamental to ensuring the clinical efficacy of the cog threads. The retention rates of tensile strength for the cog threads processed using different techniques during the degradation process are shown in Figure 8. Repeated-measures ANOVA showed that the retention of tensile strength of the three cog threads differed significantly, with a *P*-value of 0.015 in the between-subjects effects test, which falls between 0.01 and 0.05. Combined with the figure, it can be observed the tensile strength retention rates of the three types of sutures were comparable between 4 and 8 weeks. However, the cut thread exhibited a slower loss of tensile strength in the initial stage.

### Retention of Grasping Force

Facial suspension relies primarily on the barb structure of the cog threads for mechanical anchorage. After implantation into the subcutaneous tissue, the barbs interlock with the soft tissue, forming a self-tightening mechanical knot.^16^ When the thread is pulled, the barbs counteract tissue slippage through their grasping force, thereby mobilizing and gathering the soft tissues in the intended direction to achieve facial rejuvenation. As a key parameter quantifying the anchorage strength of the barbs, the grasping force directly determines the efficacy of the suspension.

In this study, the initial grasping force was 11.9 N for the injection-molded thread, 20.5 N for the compression-molded thread, and 4.6 N for the cut thread. Due to constraints imposed by the thread diameter, the cut thread with a smaller geometric profile of its barbs, resulting in its lower grasping force. Consequently, subsequent analysis and comparison were conducted between the compression-molded and injection-molded threads.

**Figure 9.**
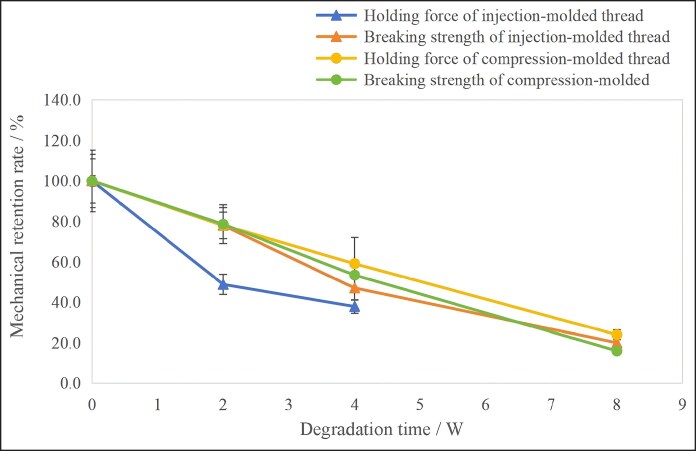
Retention of grasping force for the cog threads with different processing techniques during degradation (*n* = 6).

The results of the grasping force retention rates are presented in Figure 9. As degradation progressed, the grasping force retention rate of compression-molded thread was higher than injection-molded thread (repeated-measures ANOVA indicated that the *P*-value for the tests of between-subjects effects was 0.000).

## DISCUSSION

All PPDO samples exhibited a 2-stage degradation profile. Mass loss was slow (<10%) in the first 8 weeks, followed by a rapid degradation phase after 8 weeks, which is consistent with the study by Su.^17^ The behavior can be attributed to the high crystallinity of PPDO. In the initial degradation stage, water molecules are unable to easily penetrate the crystalline regions, leading to primary degradation in the amorphous regions. However, the molecular weight of the degradation products remains relatively high at this stage, preventing their dissolution into the degradation medium and resulting in negligible mass change. As degradation progresses to the crystalline regions, mass loss increase rapidly.^18,19^ Moreover, the degradation products cannot diffuse away from the bulk of the material, leading to a localized acidic environment that accelerates mass loss after 8 week.^20,21^ The cut threads exhibit a 2-week prolonged degradation cycle compared with the other two types of threads, which is potential to provide more prolonged stimulation for collagen regeneration in clinical applications.^5,22^ This effect is beneficial for clinical scenarios that require long-term tissue remodeling.

Specifically, the injection-molded thread exhibited the most rapid decline in intrinsic viscosity, followed by the compression-molded thread, with the cut thread degrading the slowest. This trend aligns with their distinct thermal histories during processing. The injection-molded thread experienced the highest processing temperature, as the process involves melting the polymer. The compression-molded thread was formed under pressure at a sub-melting temperature, applying moderate heat. In contrast, the cut thread was not exposed to any heat during fabrication. This validated that processing heat accelerates subsequent degradation.

The tensile strength retention rates of the three types of sutures were comparable between 4 and 8 weeks, and the mechanical properties are essentially lost at 16 weeks. In another study by Yoon,^23^ after insertion of 9 cm PPDO threads into the skin of the Yucatan pig, the authors observed newly developed fibrous connective tissue, merging with existing fibrous connective tissue, tissue contraction by myofibroblast activity, increased capillary vessel size and reduced fat layer thickness by fat cell denaturation. Additionally, they detected that the thread retains its shape for 12 weeks, which was consistent with the results of the mechanical property study in this trial. During the 26-month clinical follow-up, all 38 patients were satisfied with the facial lifting outcomes, and no other complications were observed.^24^ These findings indicate that the facial lifting effect is mainly maintained by the mechanical properties of the threads in the first 12 weeks, and after 12 weeks, it is primarily sustained by the fusion of newly formed fibrous connective tissue with native tissue.

The cut thread exhibited a slower loss of tensile strength in the initial stage. This may be attributed to the higher integrity of molecular chains in the cut thread. The room-temperature cutting process avoided thermal-induced damage to the PPDO molecular chains, thereby preserving a higher initial crystallinity and slowing the rate of water molecule penetration.^25^ As degradation progressed, however, accelerated penetration of water molecules leaded to a more substantial decline in tensile strength, aligning the behavior of the cut thread with that of the compression-molded and injection-molded threads.

**Figure 10.**
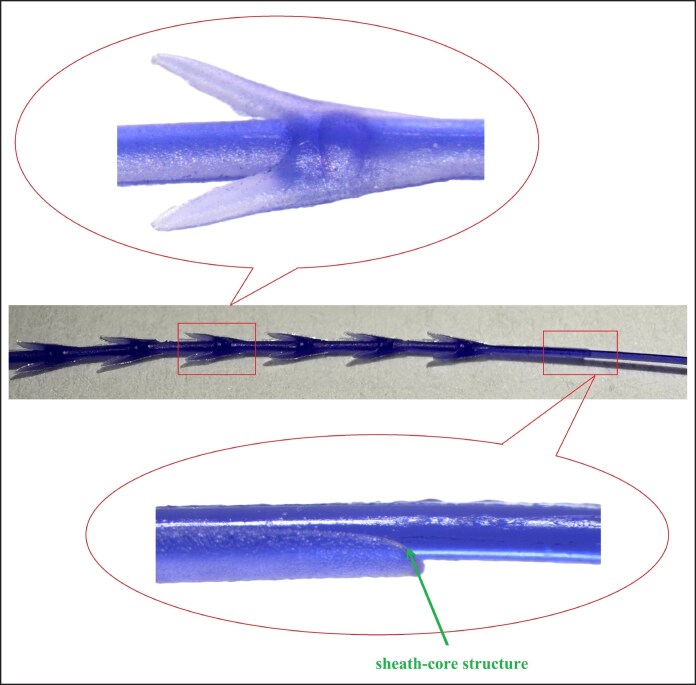
Schematic of the sheath–core structure in the injection-molded thread.

As degradation progressed, the grasping force retention rate of compression-molded thread was higher than injection-molded thread. The reason for this was that the injection-molded thread undergone a high-temperature melting during the process. The bonding interface between the barbs and the core was uneven (as shown in Figure 10). Furthermore, the barbs experienced thermal degradation during high-temperature formation, contributing to the decline in grasping force. In contrast, the compression molding process achieved integrated press-forming at a sub-melting temperature. This resulted in barbs and the thread body being a monolithic structure and induced a lower degree of thermal degradation during processing. Therefore, the compression-molded thread demonstrated a superior grasping force retention rate.

Based on the tensile strength analysis, the compression-molded and injection-molded threads showed comparable tensile strength retention rates. This was because the heating during the injection molding process primarily affected the barbs, while the core experienced minimal impact. Since the core provided the main tensile strength, both types of threads performed similarly. Further comparison revealed that the tensile strength and grasping force of the compression-molded thread decreased at nearly identical rates. In contrast, the injection-molded thread lost its grasping force more rapidly than its tensile strength. This indicated that the barb coating in the sheath–core structure were more susceptible to degradation.

**Figure 11.**
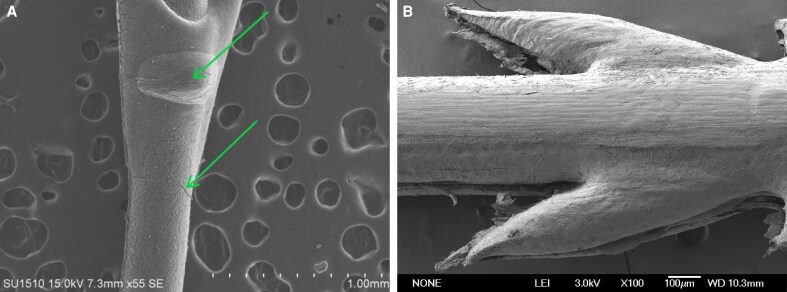
SEM images of (A) the injection-molded and (B) compression-molded threads after 8 weeks of degradation. SEM, scanning electron microscopy.

To verify the hypothesis that the barb coating in the sheath–core structure degrade more easily, we characterized both threads using scanning electron microscopy (SEM) after 8 weeks of degradation (as shown in Figure 11). The results revealed that surface cracks had developed on the barb coating of the injection-molded thread (indicated by the green arrow in Figure 11). This observation indicated a loss of structural integrity in the barb coating and a weakening bond of the sheath–core interface, which promoted barbs detachment. In contrast, only a small number of needlelike fibers were observed on the surface of the compression-molded thread, with no significant cracks detected. These microscopic morphological characteristics were consistent with the previously described mechanical behavior—specifically, the faster loss of grasping force compared to tensile strength in the injection-molded thread—and thus provided further support for the hypothesis.

To enhance the grasping force stability of the injection-molded thread, a systematic analysis of the causes behind its performance deficiencies is necessary. The relatively poor grasping force stability may primarily stem from two factors. First, the high temperature during the injection molding process induces chain scission in the polymer molecules, leading to a reduced molecular weight in the barb coating and consequently diminished mechanical properties. Second, the rapid cooling of the barb coating results in lower crystallinity, which also contributes to the decline in mechanical performance in this area. Effectively addressing these factors could further improve the mechanical properties of the injection-molded thread.

## CONCLUSIONS

This study systematically investigated the effects of different processing techniques (injection molding, compression molding, cutting) on the degradation behavior of PPDO cog threads under conditions simulating the internal human environment. The results demonstrated that the processing technique significantly influenced both the degradation rate and mechanical properties of the threads, with the following degradation trends observed:

1. Overall, all three thread types exhibited a typical two-stage degradation profile. During the first 8 weeks, mass loss was slow, while intrinsic viscosity decreased rapidly and tensile strength declined steadily. After 8 weeks, mass loss accelerated, whereas the decrease in intrinsic viscosity slowed and tensile strength was largely lost.
2. Regarding mass and intrinsic viscosity loss, the injection-molded thread, subjected to the highest processing temperature and resulting molecular chain damage, degraded the fastest, showing the most rapid rates of mass loss and viscosity reduction. In contrast, the cut thread, which experienced no thermal exposure during processing and maintained higher molecular chain integrity, exhibited a prolonged degradation cycle, making it suitable for clinical applications requiring long-term tissue remodeling. The compression-molded thread displayed intermediate characteristics.
3. Concerning mechanical performance, the cut thread showed the slowest initial decline in tensile strength due to the absence of thermal damage, though all three types eventually converged in performance. In terms of grasping force, the injection-molded thread experienced a more rapid decrease, likely attributable to thermal effects on the barbs during processing, which increased their susceptibility to detachment. The compression-molded thread demonstrated a higher grasping force retention rate, indicating superior structural stability and greater potential for maintaining long-term mechanical anchorage.

In summary, the processing technique significantly affects the degradation behavior and mechanical properties of PPDO cog threads. The appropriate selection of a processing method can effectively modulate their degradation rate and functional performance, providing a theoretical foundation and technical rationale for the clinical application of PPDO, such as in facial suspension procedures.
